# Supplementary material for: Predicting visual function by interpreting a neuronal wiring diagram
Source: Nature. 2024 Oct 2;634(8032):113–23. doi: 10.1038/s41586-024-07953-5 (PMC11446822; doi:10.1038/s41586-024-07953-5)
Supplement: Supplementary file 6 — CRF and ERF predictions for individual TmY4 and TmY9 cells. Analogous to Supplementary Data 3, but for TmY target types. Shown are the top four monosynaptic pathways, the strongest pathway passing through each of the top ten intermediary types (ranking from Extended Data Fig. 7), and the trisynaptic pathway Tm1–TmY–Dm3–TmY (see the section entitled Prediction of spatial normalization). [file 41586_2024_7953_MOESM6_ESM.zip › DataS4/TmY9qΓèÑ/720575940622084336.pdf]

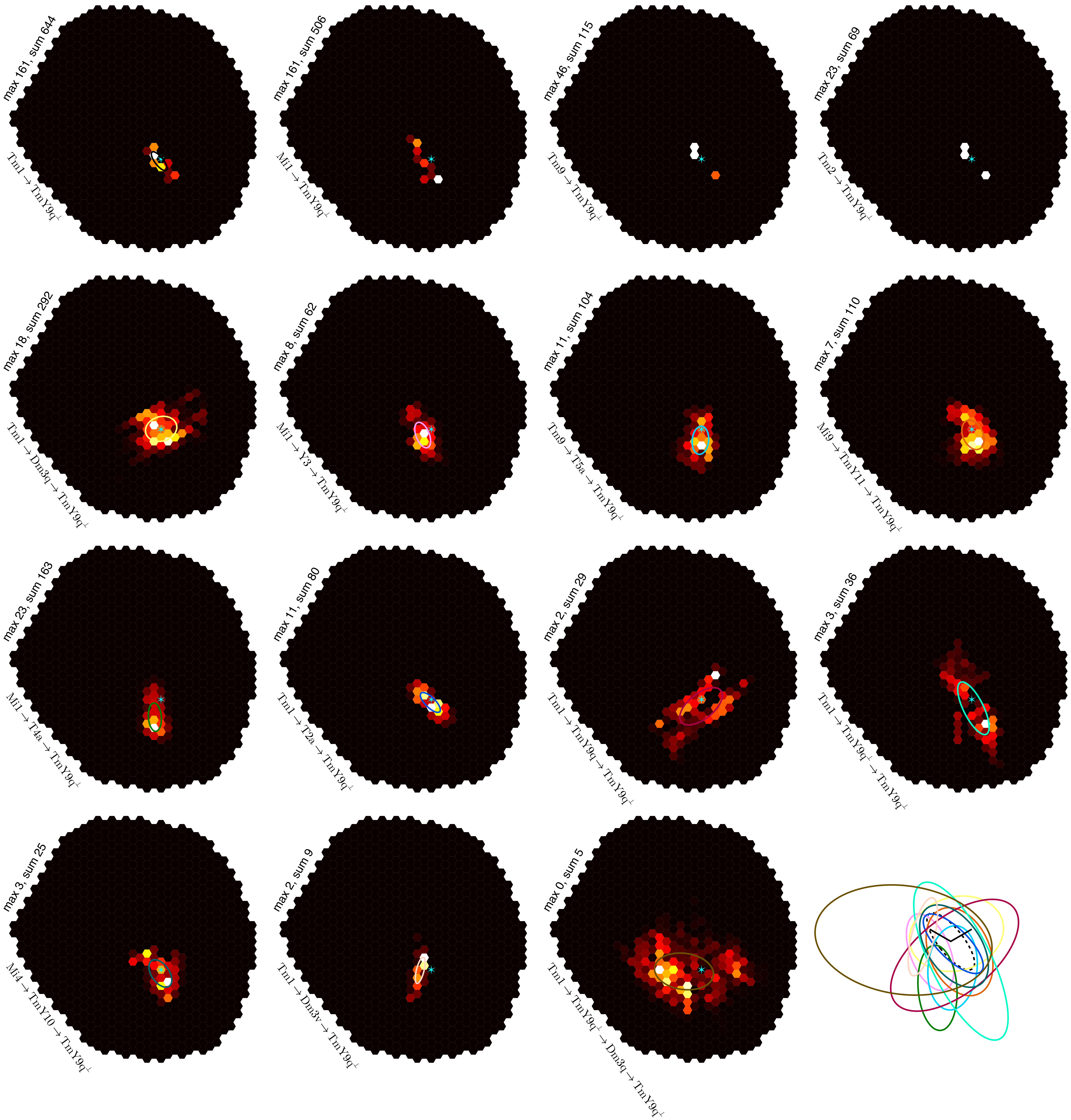
max 161, sum 644
Tm1 → TmY9q⊥
max 161, sum 506
Mi1 → TmY9q⊥
max 46, sum 115
Tm9 → TmY9q⊥
max 23, sum 69
Tm2 → TmY9q⊥
max 18, sum 292
Tm1 → Dm3q → TmY9q⊥
max 8, sum 62
Mi1 → Y3 → TmY9q⊥
max 11, sum 104
Tm9 → T5a → TmY9q⊥
max 7, sum 110
Mi9 → TmY11 → TmY9q⊥
max 23, sum 163
Mi1 → T4a → TmY9q⊥
max 11, sum 80
Tm1 → T2a → TmY9q⊥
max 2, sum 29
Tm1 → TmY9q⊥ → TmY9q⊥
max 3, sum 36
Tm1 → TmY9q⊥ → TmY9q⊥
max 3, sum 25
Mi4 → TmY10 → TmY9q⊥
max 2, sum 9
Tm1 → Dm3v → TmY9q⊥
max 0, sum 5
Tm1 → TmY9q⊥ → Dm3q → TmY9q⊥
